# Euthanasia Assessment in Ebola Virus Infected Nonhuman Primates

**DOI:** 10.3390/v6114666

**Published:** 2014-11-24

**Authors:** Travis K. Warren, John C. Trefry, Shannon T. Marko, Taylor B. Chance, Jay B. Wells, William D. Pratt, Joshua C. Johnson, Eric M. Mucker, Sarah L. Norris, Mark Chappell, John M. Dye, Anna N. Honko

**Affiliations:** 1 US Army Medical Research Institute for Infectious Diseases, 1425 Porter St., Fort Detrick, MD 21702, USA; E-Mails: john.c.trefry.ctr@mail.mil (J.C.T.); shannon.t.marko.mil@mail.mil (S.T.M.); taylor.b.chance.mil@mail.mil (T.B.C.); jay.b.wells.ctr@mail.mil (J.B.W.); william.d.pratt4.civ@mail.mil (W.D.P.); joshua.johnson@nih.gov (J.C.J.); eric.m.mucker.ctr@mail.mil (E.M.M.); sarah.l.norris2.civ@mail.mil (S.L.N.); mark.chappell@usuhs.edu (M.C.); john.m.dye1.civ@mail.mil (J.M.D.); anna.honko@nih.gov (A.N.H.); 2 Madigan Army Medical Center, 9040 Jackson Ave., Tacoma, WA 98431, USA; 3 National Institute of Allergy and Infectious Diseases, Integrated Research Facility, 8200 Research Plaza, Fort Detrick, MD 21702, USA; 4 Armed Forces Radiobiology Research Institute, 8901 Wisconsin Avenue, Building 42, Bethesda, MD 20889, USA

**Keywords:** filovirus, nonhuman primate, viral hemorrhagic fever, euthanasia, clinical pathology, Ebola virus

## Abstract

Multiple products are being developed for use against filoviral infections. Efficacy for these products will likely be demonstrated in nonhuman primate models of filoviral disease to satisfy licensure requirements under the Animal Rule, or to supplement human data. Typically, the endpoint for efficacy assessment will be survival following challenge; however, there exists no standardized approach for assessing the health or euthanasia criteria for filovirus-exposed nonhuman primates. Consideration of objective criteria is important to (a) ensure test subjects are euthanized without unnecessary distress; (b) enhance the likelihood that animals exhibiting mild or moderate signs of disease are not prematurely euthanized; (c) minimize the occurrence of spontaneous deaths and loss of end-stage samples; (d) enhance the reproducibility of experiments between different researchers; and (e) provide a defensible rationale for euthanasia decisions that withstands regulatory scrutiny. Historic records were compiled for 58 surviving and non-surviving monkeys exposed to Ebola virus at the US Army Medical Research Institute of Infectious Diseases. Clinical pathology parameters were statistically analyzed and those exhibiting predicative value for survival are reported. These findings may be useful for standardization of objective euthanasia assessments in rhesus monkeys exposed to Ebola virus and may serve as a useful approach for other standardization efforts.

## 1. Introduction

Filoviruses are highly virulent human pathogens for which no vaccines or therapeutics are currently licensed; however, several therapeutic and vaccine candidates have shown promising efficacy in nonhuman primate models of filovirus infection [1,2,3,4,5,6,7,8,9,10], and are potential candidates for use in humans against filovirus infection. Demonstrations of efficacy in nonhuman primate filovirus disease models will be central to the continued development and licensure of filovirus therapeutic and vaccine medical countermeasures, given the ethical and feasibility constraints of conducting human efficacy trials.

While various nonhuman primate filovirus infection models have been described, experimental infection of rhesus monkeys (*Macaca mulatta*) with Ebola virus has been widely used to characterize the pathophysiology of viral hemorrhagic fever and to evaluate efficacy of filovirus vaccine and therapeutic candidates. Rhesus monkeys are susceptible to infection with wild-type Ebola virus derived from human clinical isolates, through various routes of exposure, including intramuscular and respiratory [11,12,13]. A target dose of virus of 1000 plaque-forming units (PFU) has been widely used for most reported studies involving this model. While the timing of disease progression and pathophysiology vary among individual animals, manifestations of disease generally do not differ widely between animals exposed via an intramuscular *versus* respiratory routes. After exposure, initial clinical signs of illness typically develop within four to five days and include fever and reduced activity [13]. Mild alterations to clinical pathology parameters—including elevations of aspartate aminotransferase (AST), alkaline phosphatase (ALP), and/or alanine aminotransferase (ALT); and reduced lymphocyte numbers—are often observed in rhesus monkeys beginning five days after Ebola virus exposure and occur concomitantly with the incipient detection of circulating virus or viral RNA. Systemic virus titers generally increase until the time of death and disease signs often progress rapidly. Most untreated animals succumb to disease within seven to 11 days after virus exposure. Any of a number of severe alterations to serum chemistry parameters indicative of multiple organ impairment may be observed in animals preceding death (occurring either spontaneously or by euthanasia) [13,14]. These include severely elevated AST, ALP, ALT, blood urea nitrogen (BUN), creatinine (CRE), total bilirubin, and gamma-glutamyltransferase (GGT); and reduced calcium (Ca) [13]. Of the hematological parameters affected by exposure to Ebola virus, the most dramatic alterations are characterized by lymphopenia (reduced lymphocyte counts), which may rebound immediately before death), and thrombocytopenia (reduced platelets).

Interventional strategies, such as vaccination regimens or pre- or post-exposure administration therapeutic agents, can alter the timing and severity of clinical disease onset as well as the severity of alterations to clinical pathology parameters [1,5,7,15,16]. Depending on the effectiveness of the interventional strategy, rhesus monkeys infected with Ebola virus may be completely protected against infection, exhibiting no clinical signs of illness, detectable viremia, or alterations to clinical pathology parameters; or animals may exhibit reduced viremia or pathophysiology along with an extension in the time to death relative to experimental control animals. Given the high mortality rate occurring in control rhesus monkeys infected with Ebola virus, most efficacy studies utilize survival as the primary efficacy endpoint. However, it is unusual in the published literature for investigators to describe the criteria that were used to assess or justify euthanasia of an animal and in most instances the means of death (spontaneous *versus* euthanasia) for individual animals are unreported. Currently, there exists no standardized approach to assess the health status of a filovirus-infected nonhuman primate or to assess euthanasia.

To establish a standardized approach to assessing euthanasia in filovirus-infected nonhuman primates, with an emphasis in identifying objective parameters to the greatest extent possible that could be used in the euthanasia assessment, historic clinical pathology data, generated as part of therapeutic-efficacy evaluations in rhesus monkey Ebola virus hemorrhagic fever disease models, were subjected to a retrospective analysis. The goal for these analyses was to maximize both animal welfare and objective data collection in accordance with the Food and Drug Administration’s (FDA) Guide for Industry on Animal Models, the Animal Welfare Act, and the Public Health Service (PHS) Policy on Humane Care and Use of Laboratory animals [17,18,19,20]. Additionally, these specific data were identified as a critical need in the 2011 National Research Council Report on Animal Models for Assessing Countermeasures [20]. While the results discussed herein are not “the” solution, they can provide a statistical, standardized argument for endpoint assessment that will prevent loss of data and remove a certain level of subjectivity. Recommendations based on the statistical analyses for criteria to be considered for assessing euthanasia of an Ebola virus infected rhesus monkeys are provided. The approach provided in this report may be useful to guide similar euthanasia assessment strategies for other test systems or viral hemorrhagic fever disease models.

## 2. Materials and Methods

### 2.1. Animal Experiments

No animals were subjected to experimental Ebola virus infection specifically to obtain data for the analyses described in this report. All presented data were derived from historic experiments collected from ten experiments over a period of four years. Rhesus monkeys were participants in therapeutic- or vaccine-efficacy evaluations and served either as infection-control subjects or as experimental-treatment animals. Animals were exposed to a target dose of 1000 PFU of Ebola virus by intramuscular injection or by exposure to aerosolized virus. Blood samples were collected periodically during the course of infection, and in most cases, at the time of euthanasia. In all experiments, investigators euthanized animals that were observed to be moribund, as assessed using subjective clinical signs recorded at least twice daily, by pentobarbital overdose administered via intravenous injection.

Animals that survived at least 28 days after virus exposure were designated as survivors. All surviving animals reported herein were treated with an antisense-based therapeutic combination targeting Ebola virus proteins VP24 and VP35. Details of the experimental conditions that conferred protection in these animals have been described elsewhere [5].

Research was conducted under an Institutional Animal Care and Use Committee (IACUC)-approved protocol in compliance with the Animal Welfare Act, PHS Policy, and other Federal statutes and regulations relating to animals and experiments involving animals. The facility where this research was conducted is accredited by the Association for Assessment and Accreditation of Laboratory Animal Care, International and adheres to principles stated in the Guide for the Care and Use of Laboratory Animals, National Research Council, 2011.

### 2.2. Statistical Analysis

Due to variability in blood collection schedules and the retrospective nature of the evaluation, maximum and minimum laboratory values for each animal were used in the analyses. Although daily sampling was not available, MAX/MIN values were generally within 48 h of euthanasia. Dependent variables were screened for outliers, normality and homogeneity of variance. To satisfy assumptions of normality and homogeneity of variance, LOG10 transformations were applied to BUN, Creatinine, Gamma-glutamyl transpeptidase (GGT), Mean Corpuscular Hemoglobin Concentration (MCHC), and Mean Corpuscular Hemoglobin (MCH) levels. The effects of minimum and maximum laboratory values on survival outcome were assessed using a logistic regression model. Confidence intervals around odds ratios were calculated using profile-likelihood methods. Logistic regression analyses were conducted using SAS Version 9.2 (SAS Institute Inc., SAS OnlineDoc, Version 9, Cary, NC, USA, 2008). Receiver operating characteristic (ROC) curve analysis was used to determine area under the ROC curve and to evaluate sensitivity and specificity of proposed threshold values. The red line threshold on each graph was the result of the ROC curve analysis plus 10%. ROC curve analysis was conducted using SigmaPlot Version 12.5 (Systat Software Inc., San Jose, CA, USA, 2011).

## 3. Results

### 3.1. Clinical Signs of Disease

Experimental infection of nonhuman primates with Ebola virus produces a fulminate disease, characterized by a rapid reduction in animal activity and responsiveness, which necessitates that subjective assessments serve as a primary tool for assessing euthanasia needs. Five categories of clinical scores representing a combination of clinical signs that often accompany Ebola virus infection in rhesus monkeys are presented in Table 1 (while clinical categories (CC) were assigned numeric values (ranging from 0–4), it is important to note that CC designations are qualitative designations). Animals that exhibit clinical signs corresponding to CCs of 0–2 are not recommended for consideration of euthanasia because they either lack disease signs (CC 0) or because disease signs they may exhibit are relatively mild to moderate and are poorly predictive of survival outcome. For example, an animal administered an interventional treatment may exhibit disease signs associated with CC 2 transiently for one to two days before recovering, or these disease signs may progress rapidly to CC 3 or CC 4. The clinical signs described for CC 4 can be regarded as end-stage signs of terminal cases of Ebola virus hemorrhagic fever and euthanasia is strongly recommended on a humane basis for animals exhibiting the severity of disease signs described for CC 4.

**Table 1:** Assessment of Euthanasia by Subjective Clinical Signs.

Clinical Category	Clinical Signs	Prognosis	Euthanasia Assessment
0	Alert	Indeterminate	Euthanasia not recommended
	Responsive		
	Healthy		
1	Slightly diminished general activity	Indeterminate	Euthanasia not recommended
	Alert		
	Responsive		
2	Mildly unresponsive, responsive when approached	Indeterminate	Euthanasia not recommended
	Occasionally lays down		
	May exhibit hunched posture		
	Markedly Reduced Activity		
3	Moderately unresponsive (requires prodding)	Indeterminate by clinical signs	Evaluate Secondary, Objective Parameters
	Inactive, prostrate but rises when approached		
4	Severely or completely unresponsive	Poor	Euthanasia recommended
	Inactive, persistently prostrate (may momentarily rise when approached)		
	Severely labored breathing		

Animals assigned to CC 3 are animals that are moderately unresponsive to the approach of study personnel, or that only respond upon gentle physical prodding. Animals in this category may initially present in a prostrate position (*i.e.*, lying down), but may rise to an upright standing or sitting position when personnel attempt to interact with the animals from the cage exterior. While disease signs consistent with CC 3 will likely be observed for varying lengths of time in nearly all animals that ultimately succumb to infection, these signs may be transiently observed, for three or more days in certain instances, before resolving (disease resolution is typically associated with treatment with an interventional agent. To better assess the disease severity in an animal exhibiting CC 3 clinical signs, additional secondary assessments of body temperature and specific clinical pathology parameters may be useful in assessing disease severity, and ultimately in assisting with euthanasia decisions.

Other clinical signs, noted in filovirus-infected animals, including maculopapular rash, hemorrhage from bodily orifices, and convulsions, are poorly predictive of outcome (data not shown). Maculopapular rashes occur in many filovirus-infected animals, but neither the severity of the rash nor the percentage of skin affected appear to be associated with survival outcome. Typically, when bloody exudates from bodily orifices are observed, the volume of blood loss is not sufficient to warrant consideration of euthanasia, and substantial blood volume loss from hemorrhage is unusual in filovirus-infected nonhuman primates. Likewise, other severe clinical signs, such as convulsion or severe photophobia or sound sensitivity, present infrequently. The infrequent occurrence of these clinical signs diminishes their contribution toward a generalized approach for euthanasia assessment.

### 3.2. Body Temperature

Body temperature in filovirus-infected nonhuman primate assessments may be measured in biosafety level-4 research labs using automated telemetric monitoring of core-body temperature or, in sedated animals, by rectal thermometer. When assessed using real-time telemetric monitoring, circadian fluctuations of core-body temperature of 1–2 °C are readily apparent in graphical displays. During the late-stage of terminal Ebola virus infections in rhesus monkeys, decrease in core body temperature results from failure of thermoregulatory mechanisms that are likely secondary to the insult on essential physiological processes required to sustain life.

The rectal body temperature of healthy rhesus monkeys ranges from 37.0–39.5 °C [21]; however, single point temperature assessments can provide misleading baseline values given the fluctuations that accompany circadian rhythmic cycles. Rectal temperatures of 34 °C or less may be considered indicative of terminal-stage collapse of thermoregulatory mechanisms in Ebola virus infected rhesus monkeys, especially if animals concomitantly exhibit clinical signs consistent with CC 3 (because of the hypothermic effects of sedation, it is critical that any temperature assessment be conducted immediately following sedation). In animals that are implanted with telemetric devices to monitor core temperature, animals displaying CC 3 clinical signs and which have a temperature less than 4 °C below the animal’s baseline are likely undergoing thermoregulatory collapse. Baseline telemetry data were acquired for each animal over several days to generate hourly baseline temperatures in order to account for each animal’s diurnal rhythm. Animals exhibiting CC 3 clinical signs in combination with a rectal temperature <34 °C or a deviation from baseline temperature of ≥4 °C (for telemetrically monitored animals) are recommended for euthanasia.

The authors acknowledge that sedation of an animal exhibiting multiple clinical signs of Ebola virus hemorrhagic fever introduces a stressor that may exacerbate the disease and potentially hasten the animal’s death, e.g. if the animal fails to adequately recover from sedation or if sedation interferes with food and water consumption. However, other less invasive means of temperature acquisition, e.g., infrared thermal thermometers, to record surface body temperature may provide inaccurate values because measurements must be obtained through stainless steel cage bars. These methods would have to be evaluated and validated by other investigators at their research sites.

### 3.3. Clinical Pathology

The proposed euthanasia-threshold temperature values are intended to identify animals that are in an irreversible, terminal stage of disease progression and that, without euthanasia intervention, are likely to spontaneously succumb within hours. However, in many instances, animals exhibiting CC 3 signs will not exhibit reduced body temperatures. To evaluate whether alterations of routine clinical pathology parameters may be useful in euthanasia assessments, historic biological data from 58 rhesus monkeys that either survived or succumbed to Ebola-virus infected were compiled and subjected to retrospective analysis.

#### 3.3.1. Dataset Summary

Animals were infected by either intramuscular injection (*n* = 52) or exposure to aerosolized virus (Table 2). All aerosol-infected animals were infection-control subjects and succumbed or were euthanized seven to nine days after virus exposure. Of those animals exposed by the IM route, 13 were infection-control subjects and 12 succumbed or were euthanized seven to 11 days after virus exposure, although one IM-infected control animal survived until scheduled termination of the study 33 days after infection (a description of clinical pathology alterations observed in this atypical surviving control animal is described elsewhere [13]). Of the 39 IM-infected animals that were treated with one of several therapeutic agents, 12 survivors were reported, all of which were treated with a combination of antisense agents (positively charged phosphorodiamidate morpholino oligomers) targeting Ebola virus proteins VP24 and VP35. Details of these surviving animals and the treatment conditions that facilitated their survival have been described elsewhere [5]. Mean time-to-death for IM-infected control animals was 8.8 days (range seven to 11 days). Animals that were treated with a therapeutic agent but which ultimately succumbed to infection (euthanized) exhibited a mean time-to-disposition of 9.9 days, with a range of seven to 17 days.

Because of the retrospective nature of this analysis, the available data varied depending on individual study designs. To assess whether clinical pathology alterations were related to survival outcome, maximal and minimal values of each clinical pathology parameter, obtained from an individual animal at any time during the course of infection, regardless of the sampling schedule, were subjected to logistic regression analysis. Sixteen parameters exhibited a suggested relatedness to survival outcome (Table 3). Additional parameters that did not show a significant correlation to survival are listed in Supporting Information. Alterations to seven clinical chemistry parameters (BUN, CRE, total bilirubin, total calcium, GGT, amylase, and glucose) and to six hematology parameters (platelets, lymphocytes, MCH, MCHC, hemoglobin, and WBC) exhibited significant relatedness to survival.

Analysis of both hematology and clinical chemistry parameters requires acquisition of two blood samples. To reduce stress to the animal, it is desirable to minimize the volume of blood collected as part of the euthanasia assessment. Serum chemistry assessment may provide data that is more indicative of the various physiological disturbances that are likely to be affected in nonhuman primates during the course of Ebola virus infection than hematology assessment. Blood chemistry analyses can be rapidly conducted with a relatively small sample volume, and most BSL-4 laboratories are equipped with modern clinical chemistry analytical instrumentation. Therefore, this remainder of this report focuses on clinical chemistry parameters that may be useful when assessing euthanasia.

**Table 2:** Summary of Experimental Conditions and Survival Outcome for Ebola-Virus Infected Rhesus Monkeys.

Experimental Conditions	Total Number of Animals	Number of Survivors	Number of Non-Survivors	Number of Males	Number of Females	Mean Time to Disposition for Non-Survivors (days)	Range of Time To Disposition (days)
IM Infections							
Infection-control	13	1	12	5	8	8.8	7–11
Therapeutic treatment	39	12	27	25	14	9.9	7–17
Aerosol Infections							
Infection-Control	6	0	6	1	5	8.2	7–9
Therapeutic treatment	0	-	-	-	-	-	-

**Table 3:** Results of logistic regression analysis of maximal or minimal clinical pathology parameters (assessed over the course of Ebola virus infection) and survival outcome in 58 rhesus monkeys. Data were compiled from multiple independent experiments. Parameters exhibiting a significant relatedness to survival outcome (*p*-value < 0.10) are presented.

Variable	N (Survivors)	N (Non-survivors)	Odds Ratio (95%CI)	*p*-value
MAX BUN (log-transformed)	13	42	43.0 (4.51, 703)	0.0029
MAX Platelet (log-transformed)	13	45	<0.001 (<0.001, 0.013)	0.0042
MAX Creatinine (log-transformed)	13	42	64.7 (5.09, >999)	0.0054
MAX Lymphocytes	13	45	0.896 (0.818, 0.960)	0.0064
MAX Total bilirubin (log-transformed)	13	42	410 (8.66, >999)	0.0117
MIN Total calcium	13	42	0.376 (0.149, 0.744)	0.0143
MAX MCH (log-transformed)	13	45	>999 (>999, >999)	0.0258
MIN MCH (log-transformed)	13	45	>999 (556, >999)	0.0309
MAX GGT (log-transformed)	12	37	17.1 (1.33, 377)	0.0438
MAX MCHC (log-transformed)	13	45	>999 (175, >999)	0.0520
MIN MCHC (log-transformed)	13	45	>999 (205, >999)	0.0520
MAX Amylase (log-transformed)	12	37	0.009 (<0.001, 0.898)	0.0590
MAX Hemoglobin	13	45	1.82 (1.05, 3.85)	0.0685
MIN Glucose	13	42	0.984 (0.883, 0.996)	0.0733
MAX WBC (log-transformed)	13	45	0.047 (<0.001, 1.44)	0.0872
MIN GGT (log-transformed)	12	37	183 (0.493, >999)	0.0952

#### 3.3.2. Analysis of Clinical Pathology Parameters

**Figure 1:**
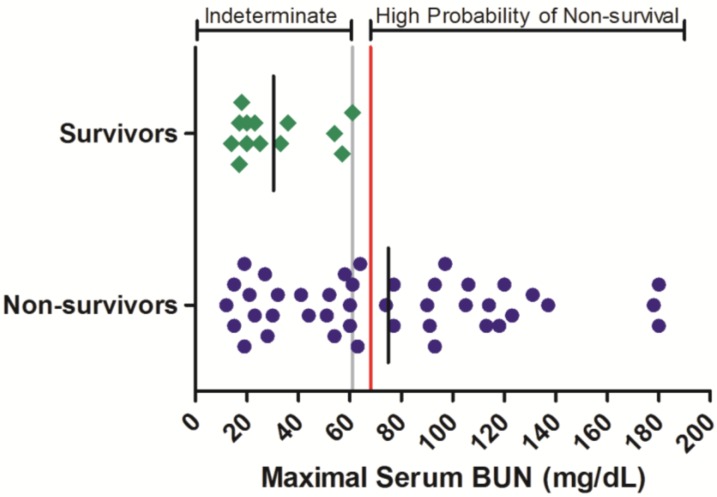
Serum BUN responses in rhesus monkeys exposed to Ebola virus. Symbols represent the maximal serum BUN value obtained at any time during the course of infection for an individual animal (diamonds = survivors, circles = non-survivors). Data were compiled from multiple independent historic experiments. The horizontal gray line indicates a survival threshold, *i.e.*, the maximal value obtained in any animal that survived infection. The red line indicates a proposed euthanasia threshold value based on ROC curve suggested values plus 10%. Black lines are the group mean. Normative serum BUN reference range for rhesus monkeys (mean +/− 1 SD) is 15–21 mg/dL [21].

Azotemia, elevations to BUN and serum CRE, is often observed in filovirus-infected nonhuman primates [13]. To assess whether the severity of azotemia is related to survival outcome in Ebola virus infected rhesus monkeys, historic results from time-course BUN and CRE serum assessments were obtained for 55 animals, of which 13 survived (Figure 1). Maximal serum BUN values ranged from 12 to >180 mg/dL in animals that succumbed to infection, while in surviving animals, the greatest maximal serum BUN observed was 61 mg/dL. Of the 42 non-survivors for which BUN was monitored, 22 (52%) of these exhibited a maximal BUN value exceeding 61 mg/dL before or at the time they succumbed to infection or were euthanized. While serum BUN values < 61 mg/dL were poorly predictive of survival outcome, increased serum BUN values were significantly related to an increased probability of non-survival (OR = 43.0 (3.62, 511); *p* < 0.05; Table 3).

**Figure 2:**
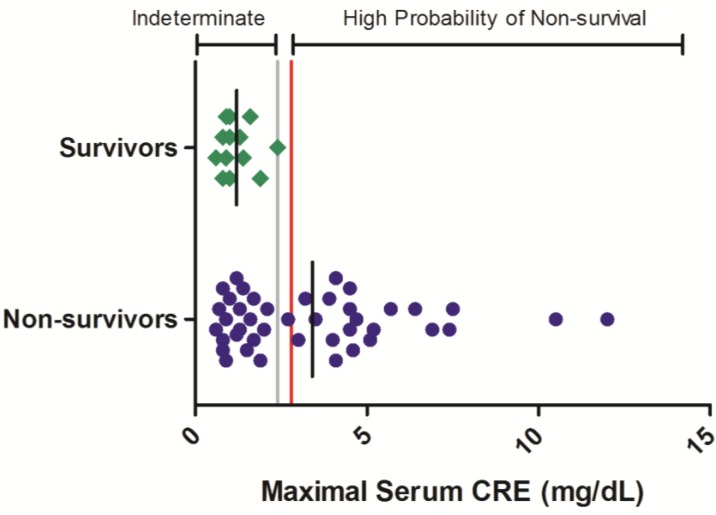
Serum CRE responses in rhesus monkeys exposed to Ebola virus. Symbols represent the maximal serum CRE value obtained at any time during the course of infection for an individual animal (diamonds = survivors, circles = non-survivors). Data were compiled from multiple independent historic experiments. The horizontal gray line indicates a survival threshold, *i.e.*, the maximal value obtained in any animal that survived infection. The red line indicates a proposed euthanasia threshold value based on ROC curve suggested values plus 10%. Black lines are the group mean. Normative serum CRE reference range for rhesus monkeys (mean +/− 1 SD) is 0.45–0.71 mg/dL [21].

Maximal CRE values ranged from 0.8 to >12.0 mg/dL in non-surviving animals while in surviving animals, no serum CRE value exceeded 2.4 mg/dL (Figure 2). In the 42 non-survivors included in this analysis, 52% exhibited a maximal serum CRE exceeding 2.4 mg/dL on at least one sampling event during the course of disease. Thus, while serum CRE values < 2.4 mg/dL offer limited predictability of survival outcome, greater CRE values are significantly related to a high probability of mortality (OR = 64.7 (3.43, 999); *p* < 0.05; Table 3).

**Figure 3:**
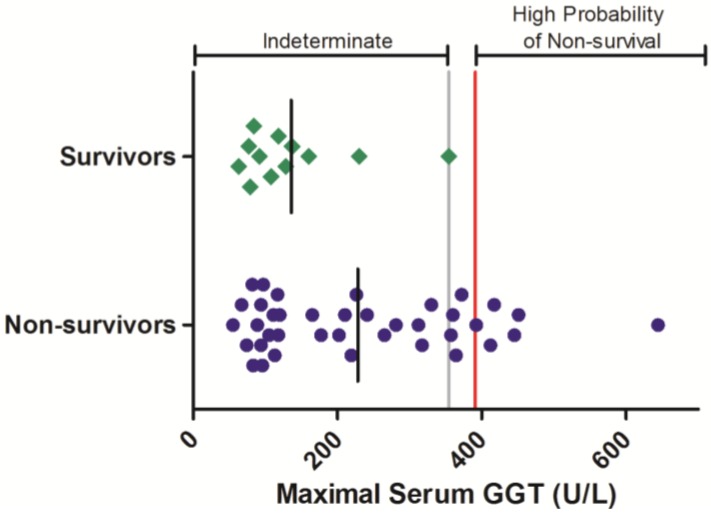
Serum GGT responses in rhesus monkeys exposed to Ebola virus. Symbols represent the maximal serum GGT value obtained at any time during the course of infection for an individual animal (diamonds = survivors, circles = non-survivors). Data were compiled from multiple independent historic experiments. The horizontal gray line indicates a survival threshold, *i.e.*, the maximal value obtained in any animal that survived infection. The red line indicates a proposed euthanasia threshold value based on ROC curve suggested values plus 10%. Black lines are the group mean. Normative serum GGT reference range for rhesus monkeys (mean +/− 1 SD) is 66–97 U/L [21].

Gamma-glutamyl transferase is another blood chemistry parameter that is often elevated in response to Ebola virus infection in nonhuman primates. The serum activity of this enzyme was evaluated periodically during Ebola-virus infection in 50 animals, comprised of 12 survivors and 38 non-survivors (Figure 3). In animals that survived infection, individual maximal GGT activities were not observed to exceed 354 U/L. Maximal GGT exceeding this level were observed in 10 animals that succumbed to infection. Logistic analysis shows that elevations to GGT are significantly related a negative survival outcome (OR = 17.1 (1.03, 269); *p* < 0.05; Table 3).

**Figure 4:**
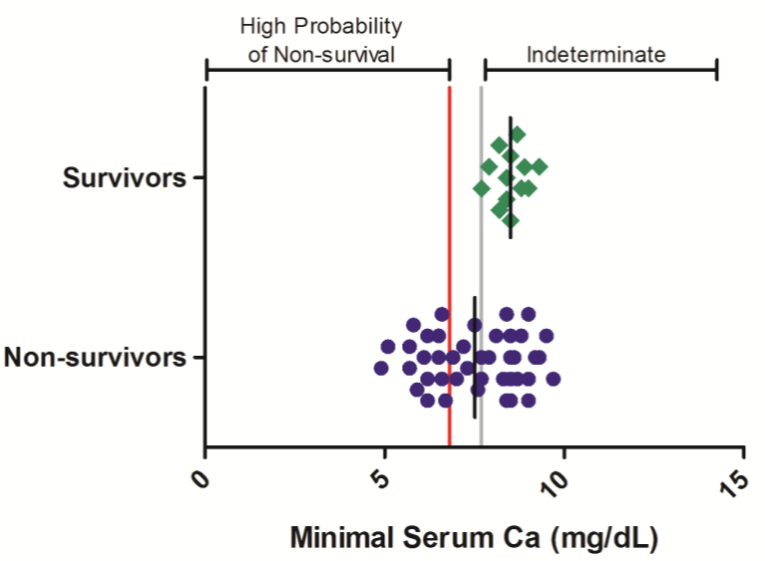
Serum total calcium responses in rhesus monkeys exposed to Ebola virus. Symbols represent the minimal serum total calcium value obtained at any time during the course of infection for an individual animal (diamonds = survivors, circles = non-survivors). Data were compiled from multiple independent historic experiments. The horizontal gray line indicates a survival threshold, *i.e.*, the maximal value obtained in any animal that survived infection. The red line indicates a proposed euthanasia threshold value based on ROC curve suggested values plus 10%. Black lines are the group mean. Normative serum total calcium reference range for rhesus monkeys (mean +/− 1 SD) is 8.6–9.2 mg/dL [21].

In contrast to BUN, CRE, and GGT, total serum calcium levels are often observed to decrease relative to baseline values in filovirus-infected nonhuman primates. Data from historic evaluations, in which total serum calcium was assessed at various times during Ebola virus infection, were analyzed in 55 rhesus monkeys, composed of 13 surviving and 42 non-surviving animals (Figure 4). In surviving animals, minimal serum calcium values were not observed to decrease below 7.7 mg/dL, whereas in nonsurvivors, minimal serum calcium values of < 7.7 mg/dL were observed in 21 of 42 (50%) of animals during at least one sampling event. Logistic regression analysis of these results show that severe hypocalcemia is significantly related (*p* < 0.05) to the probability of nonsurvival in the Ebola virus rhesus monkey disease model (OR = 0.376 (0.172, 0.823); *p* < 0.05; Table 3).

## 4. Discussion

**Figure 5:**
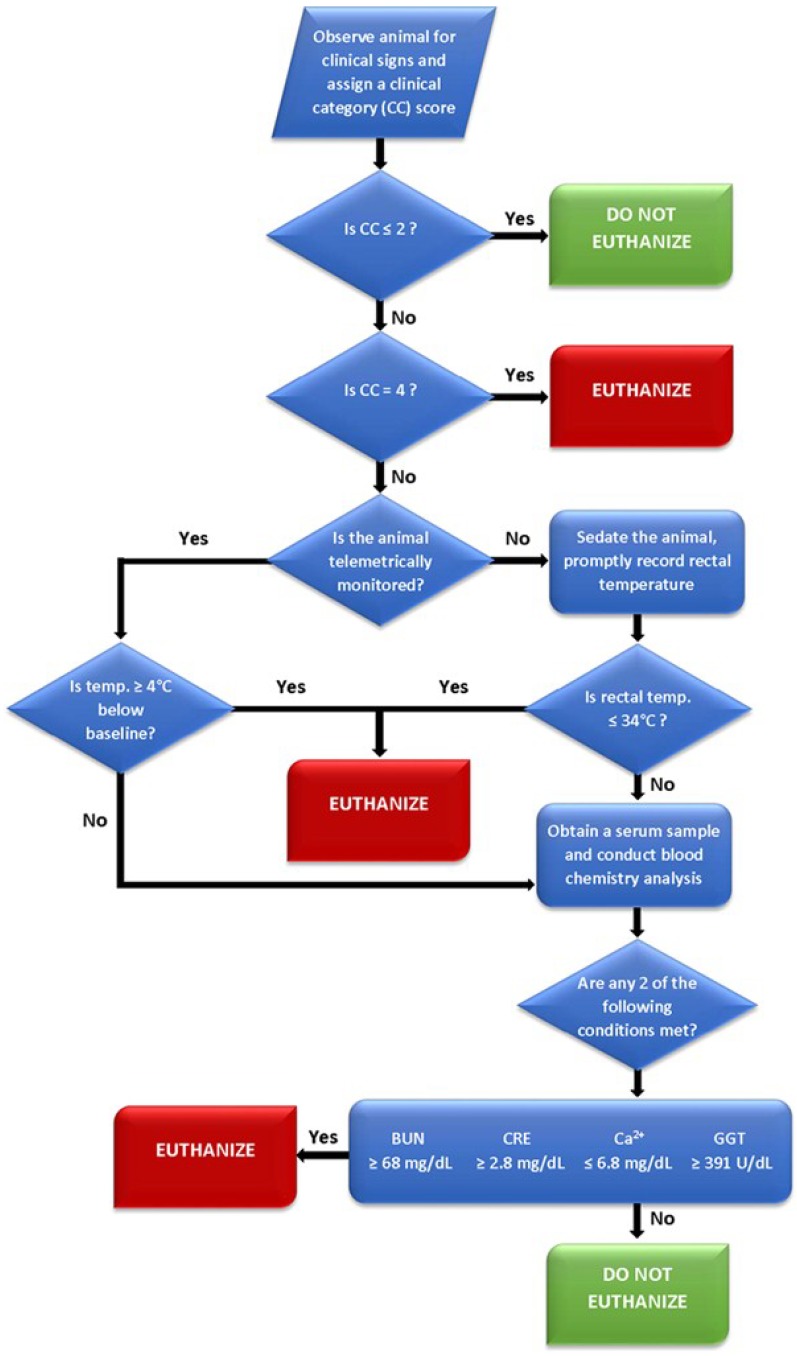
Flow diagram depicting a proposed euthanasia decision tree for Ebola virus infected rhesus monkeys.

Results obtained from the logistic regression analysis of time-course clinical pathology parameters obtained from historic evaluations suggest that extreme alterations to several commonly monitored serum parameters—specifically BUN, CRE, GGT, and total calcium—are related to survival outcome. Consideration of extremes in these serum parameters, in context with clinical observations and body temperature assessment, may be a useful approach to ensure that animals with a poor survival prognosis are identified as early as possible and humanely euthanized. One approach to how these parameters could be considered when assessing euthanasia is presented in Figure 5.

The CC designations are the primary evaluation upon which secondary criteria (*i.e.*, body temperature and clinical chemistry values) may then be assessed. The 0–4 scoring system was the product of distilling multiple euthanasia criteria into their lowest common denominators and eliminating some of the potential biases posed by compounding multiple subjective assessments (*i.e.*, a point system for observations, such as coat condition, dehydration, appetite, *etc.*). By progressing to these secondary criteria at a designation of CC 3, an investigator can then base decisions on objective measurements to accurately gauge the need for humane intervention. Additionally, assigning a CC designation prior to evaluating secondary criteria will prevent transient anomalies in temperature, such as a drop during anesthesia, from impacting endpoint decisions. If secondary euthanasia criteria are assessed, either 1) a temperature ≥4 °C from baseline (telemetry) or ≤34 °C will result in euthanasia or 2) at least two of the clinical chemistry parameters are met will result in euthanasia.

Several key factors were considered in developing the proposed euthanasia assessment strategy. Because clinical signs may rapidly deteriorate in Ebola virus infected animals, the assessment process must be capable of being rapidly executed. Additionally, because blood will typically have been collected at multiple time points prior to euthanasia assessment, any criteria that depend on collection of a blood specimen should minimize the blood volume collected to avoid exceeding blood-volume collection limits. Further, the proposed euthanasia assessment minimizes a reliance on baseline or other animal-specific biological data, which may not be immediately accessible to technical staff in the BSL-4 laboratory.

The euthanasia-decision thresholds associated with each clinical pathology parameter were directly derived from the survival-threshold values representing the single greatest maximal (BUN, CRE, and GGT) or least minimal (total calcium) value obtained from any surviving animal at any time during infection. Euthanasia-decision thresholds were established at a value 10% greater than the survival-threshold for parameters that are elevated during Ebola virus infection and 10% lower than the survival-threshold for parameters that are decreased during infection to reduce the likelihood that implementation of these euthanasia criteria does not result in the premature euthanasia of an animal which may ultimately recover. At the level of the greatest maximal or least minimal in a surviving animal, the positive predictive value (PPV) for the parameters is as follows: BUN (100%); CRE (100%); GGT (96%); Ca (94%). With the added buffer of approximately 10%, the PPV for all parameters becomes 100%. The analysis of existing and future data from additional surviving animals will permit the refinement of survival- and euthanasia-decision thresholds.

The euthanasia decision tree proposed herein is meant to be a jumping off point for an evolving set of criteria adaptable to the circumstances facing each institute and its researchers; in fact, glucose was initially included in these criteria but was ultimately dropped. In addition to concerns about anesthesia affecting blood glucose concentration, accurate glucose measurements require precise timing when blood draws are not performed in a standard fluoride tube; however, limited sample volumes for serially drawn NHPs make the use of multiple specialty tubes impractical due to the number of downstream assays for which they are compatible. In order to minimize any potential impact posed by the time constraints of performing blood analysis across the spectrum of biosafety laboratories at USAMRIID, it was decided to drop glucose as a secondary euthanasia parameter. Despite these difficulties there is a definite downward trend for glucose values during late stages of filoviral hemorrhagic fever and as a result additional statistical analysis of glucose as a secondary criterion for euthanasia is ongoing.

An expected effect, and potential caveat, of implementing a euthanasia-assessment strategy that relies on clinical pathology determinants is that model-specific time-to-disposition may decrease relative to historic records. This would result from the euthanasia of animals that present with a clinical profile that satisfies euthanasia criteria, but which historically may not have triggered euthanasia based solely on clinical signs. Differences in time-to-disposition resulting from the manner in which euthanasia is assessed may be a particularly relevant consideration as the performance of new therapeutic agents and vaccine candidates are compared with the historic results. Additionally, other therapeutic agents may produce different values for the clinical parameters being measured herein. However, identifying objective markers that are related to a negative survival outcome will allow researchers to minimize pain and distress through euthanasia in animals that are unlikely to survive. Standardizing euthanasia criteria will provide greater defensibility of survival results, and will enhance the confidence with which survival statistics obtained by different research teams can be compared. Additionally, introducing objective criteria into euthanasia decisions reduces the emotional toll on scientific personnel required to conduct this ethically challenging process. Overall, the benefits of consistency of the model outweigh the possible effect on time to disposition and will maximize the utility of the data produced by enhancing the efficient use of a scarce resource.

The pathophysiologic disturbances responsible for the extreme clinical pathology alterations often observed in filovirus-infected nonhuman primates are not well understood. Azotemia, detected as increased BUN and/or CRE, may be caused by pre-renal and/or renal conditions [22]. In Ebola virus infected nonhuman primates, azotemia may result from pre-renal conditions such as a reduction in the glomerular filtration rate due to dehydration and/or hypovolemia, or from renal conditions, such as direct renal damage resulting from inflammatory responses to viral infection. Increases of serum GGT, a cell-membrane associated protein present on many cell types, may be indicative of cholestasis (especially when concomitant ALP elevations are observed) or of damage to renal tubular epithelial cell [22]. Cholestasis is the reduction of bile flow or excretion with many intrahepatic and extrahepatic causes. In filovirus-infected nonhuman primates, cholestasis is likely secondary to intrahepatic obstruction of bile flow from inflammation associated with viral infection of the liver. Findings of hypoglycemia may be indicative of end-stage hepatic failure and/or intestinal malabsorption. While weight loss and reduced food intake are often observed in filovirus infected NHP, lack of food intake is not usually considered a differential for hypoglycemia: fasting and anorexic animals are typically able to maintain serum glucose levels within reference limits [23]. Hypocalcemia may be secondary to hyopalbuminemia (although serum albumin in Ebola-virus infected animals was not significantly related to survival outcome as assessed by logistic regression analysis) and/or renal disease, perhaps due to ischemia [22].

Although the pathophysiologic disturbances that lead to extreme clinical pathology alterations during Ebola virus infections of rhesus monkeys are not completely understood, clinical pathology alterations observed in this model are often reported in other nonhuman primate infection models. This stands to reason as other filoviruses, e.g., Marburg virus and Sudan virus, produce a fulminate disease in various nonhuman primate test systems that is clinically difficult to distinguish from that of Ebola virus infection. Though the euthanasia assessment criteria described herein was derived from data generated from Ebola virus infected rhesus monkeys and survivors from one therapeutic platform, these criteria and/or the approach of retrospectively analyzing clinical pathology data from historic surviving and non-surviving animals has been successfully utilized for other Ebola studies, and may prove useful for refining euthanasia-decision criteria for other filovirus-infection test systems or viral hemorrhagic fever disease models.

## Supplementary Files

Supplementary File 1
